# Hydrogen-Induced Dislocation Nucleation and Plastic Deformation of 〈001〉 and $\langle 1\bar{1}0\rangle$ Grain Boundaries in Nickel

**DOI:** 10.3390/ma15186503

**Published:** 2022-09-19

**Authors:** Jiaqing Li, Ziyue Wu, Lin Teng, Guanyu Deng, Rui Wang, Cheng Lu, Weidong Li, Xin Huang, Yu Liu

**Affiliations:** 1 College of Chemical Engineering, Fuzhou University, Fuzhou 350116, China; 2 School of Mechanical, Materials, Mechatronic and Biomedical Engineering, University of Wollongong, Wollongong, NSW 2522, Australia; 3 School of Mechanical Engineering, Nantong University, Nantong 226019, China

**Keywords:** molecular dynamics, hydrogen embrittlement, dislocation nucleation, plastic deformation

## Abstract

The grain boundary (GB) plays a crucial role in dominating hydrogen-induced plastic deformation and intergranular failure in polycrystal metals. In the present study, molecular dynamics simulations were employed to study the effects of hydrogen segregation on dislocation plasticity of a series of symmetrical tilt grain boundaries (STGBs) with various hydrogen concentrations. Our study shows that hydrogen both enhances and reduces dislocation nucleation events from STGBs, depending on different GB structures. Specifically, for 〈001〉 STGBs, hydrogen does not affect the mode of heterogeneous dislocation nucleation (HDN), but facilitates nucleation events as a consequence of hydrogen disordering the GB structure. Conversely, hydrogen retards dislocation nucleation due to the fact that hydrogen segregation disrupts the transformation of boundary structure such as Σ9 ($2\;2\;\bar{1}$) $\langle 1\bar{1}0\rangle$ STGB. These results are helpful for deepening our understanding of GB-mediated hydrogen embrittlement (HE) mechanisms.

## 1. Introduction

As it is light, storable, energy-dense, and produces no direct emissions of pollutants or greenhouse gases, hydrogen has emerged as a critical pillar in any aspiring global net zero path. According to Goldman Sachs, hydrogen energy could develop into a market worth over $1 trillion a year [1]. However, the transportation and storage systems such as high-pressure pipelines, long-tube tailers, and vessels typically suffer from hydrogen embrittlement (HE) [2], severely obstructing hydrogen applications.

In the early 1870s, the H atom was firstly proposed to have damaging effects on iron and steel [3]. Since then, significant efforts have been made to characterize and comprehend the HE phenomenon of materials. Overall, the HE is a failure mechanism associated with the loss of plasticity caused by the presence of H in materials. The H atom may enter the material during production procedures such as welding or electroplating, namely called “internal hydrogen embrittlement” (IHE), or it can enter the material from the surrounding environment during service exposure, known as “environmental hydrogen embrittlement” (EHE) [3,4,5]. Solute H occupies and diffuses across interstitial lattice sites in metals and can be trapped to different degrees at defects [3,6]. So far, many HE mechanisms have been proposed. The most explained embrittlement mechanisms are hydrogen-enhanced decohesion (HEDE) [4,7,8,9] and hydrogen-enhanced localized plasticity (HELP) [10,11,12,13,14]. According to the HEDE mechanism, the decohesion occurs on account of metal charge transfer and weakening of interatomic bonds so that tensile separation of atoms occurs in preference to slip [5]. This mechanism can explain how the HE causes intergranular cracking along grain boundaries (GBs) [15]. In addition, the HELP mechanism was proposed to explain the quasi-cleavage feature, which suggests that H atom enters and concentrates on the crack tip, and deformation occurs at the crack tip due to H-promoted dislocation motion [10,14,16]. Although these mechanisms are distinct in nature, they are not mutually exclusive and can frequently occur simultaneously, depending on materials, microstructures, and environmental factors.

GBs significantly affect the transport and segregation of H and fracture mechanisms in polycrystalline materials. The GB is more favorable for H adsorption than the interstitial lattice sites. The local critical H concentration in metals is the most crucial factor communed by all HE mechanisms. Therefore, to reach the critical H concentration, H atoms must be able to diffuse rapidly into the GBs and be efficiently trapped there. Numerous studies [17,18,19,20,21] have proven that the presence of geometrically required dislocations (GNDs) accelerates H diffusion along GBs. However, Curtin [22] showed that special GBs could trap H atoms and reduce H diffusion. H atoms trapped by GBs not only change the interaction between dislocations and GBs but also change the cleavage fracture along GBs [23]. Recent studies based on the observation of microstructures beneath the fracture features have shown that the presence of H changes the evolution of the dislocation structure. The fracture mode may develop from transgranular to intergranular [24]. Therefore, studies of HE mechanisms and damage have focused on H-GB interactions.

Due to the low concentrations and great diffusivity of H in engineering alloys, HE phenomena have historically been difficult to be analyzed using conventional experimental approaches. Recent advancements in experimental techniques such as atom probe tomography [25] and neutron tomography [26] have permitted direct microstructural investigations of H segregation. However, these experimental devices are complex to customize and difficult to implement. Consequently, numerous simulation-based studies have been developed to improve our understanding of HE in metals [27,28,29,30,31,32,33]. For example, Liang et al. [34] proposed that H accelerates dislocation microstructure evolution through a H-enhanced plasticity mechanism, and the interaction of dislocations with GBs can change the ultimate failure process. Li et al. [35] found that boundary disruption and the concentration of local stress states on the GB through plastic processes promote the embrittlement effect of H atoms in metallic materials. Therefore, simulation-based techniques play an important role in unveiling more details about HE in metals.

The present study aims to explore the dislocation nucleation and plastic deformation mechanisms of GBs tilted along 〈001〉 and $\langle 1\bar{1}0\rangle$ axes in nickel with different H concentrations using molecular dynamics (MD) simulations. The content of the present study is organized as follows: the simulation method is introduced in Section 2, the GB structure, hydrogen segregation map and dislocation nucleation mechanisms are presented and discussed in Section 3, and our conclusions are summarized in Section 4.

## 2. Materials and Methods

**Figure 1:**
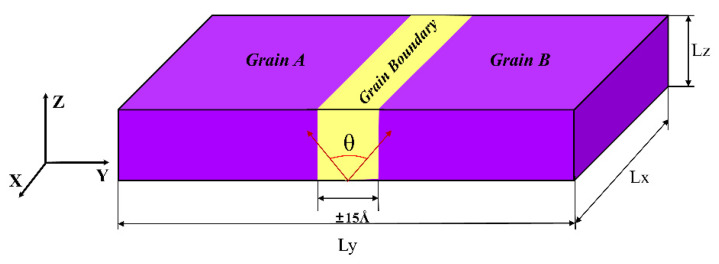
Schematic illustration of the STGB with tilt axis along the Z direction and the normal to the boundary plane along the Y direction.

The established bicrystal model with symmetric tilt grain boundary (STGB) is shown in Figure 1. The Z axis is defined as the tilt axis (〈001〉 and $\langle 1\bar{1}0\rangle$). The STGB was respectively constructed by rotating grain A and grain B with $\pm \frac{\theta }{2}$ along the tilt axes, forming a symmetrical tilt boundary. Table 1 presents the information of all chosen simulation models. The simulation model applied periodic boundary conditions along all directions, and the length of the model in the X direction and Y direction should be enough to minimize the image force.

In the present study, all MD simulations were performed using large-scale atomic/molecular massively parallel simulator (Lammps) software [36] with the embedded-atom method (EAM) interatomic potentials for Ni-H developed by Angelo et al. [37]. The Voronoi tessellation of atoms was constructed with the Voro++ code to find possible hydrogen trapping sites in each GB structure [38].

The local *H* concentration at the GBs is represented by the following equation [39]:

(1) $$C_{H}=\frac{N_{H}}{A}$$

where A is the cross-sectional area of the GB, and $N_{H}$ is the number of *H* atoms in the region.

The GB energy is defined as:

(2) $$E_{GB}=\frac{E_{region}-E_{atom}N_{atom,Ni}}{A}$$

where $E_{region}$ is the GB energy of *Ni* atoms in the region ±15 Å above and below the GB, the cohesive energy of each *Ni* atom in bulk is designated as $E_{atom}$, $N_{atom,Ni}$ is the number of Ni atoms in the selected region.

MD simulations were performed under the isothermal–isobaric ensemble (NPT) for model with and without H, respectively. The Nose-Hoover method [40,41] was used to keep the system temperature at 10 K. The integration time step of MD was set to 0.001 ps, and the deformation was achieved by stretching the box dimensions and then averaging the displacement over each atom at a strain rate of 10^−8^ s^−1^. Crystal structures were characterized by common neighbor analysis (CNA) [42] and centro-symmetry parameter (CSP) [43], and dislocation types were identified by the dislocation extraction algorithm (DXA) [44] in the Open Visualization Tool (OVITO) [45].

## 3. Results and Discussion

### 3.1. GB Energy and GB Structure

**Figure 2:**
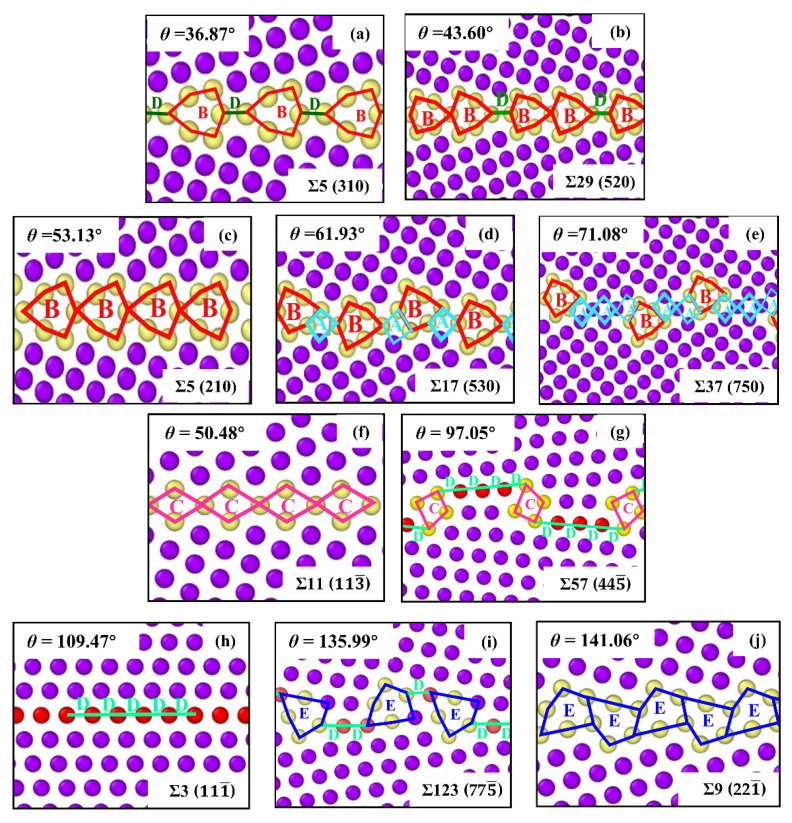
The atomistic configurations of several GB structure units along (**a**–**e**) 〈001〉 tilt axis and (**f**–**j**) $\langle 1\bar{1}0\rangle$ tilt axis. Images are colored by CNA, where the atoms in FCC structure are colored in purple, the atoms in HCP structure are colored in red, the atoms in other structure are colored in yellow.

The GB structure described by the structural unit model (SUM) is shown in Figure 2. The Σ5 (2 1 0) STGB is composed of only B structural unit (SU), and the ratio of B SU decreases with an increase in the misorientation angle, as presented by Σ17 (5 3 0) and Σ37 (7 5 0) STGBs in Figure 2d,e. For the three $\langle 1\bar{1}0\rangle$ tilted bicrystals with STGBs of Σ11 ($1\;1\bar{\;3}$), Σ3 ($1\;1\;\bar{1}$) and Σ9 ($2\;2\;\bar{1}$), the GB contains exactly *C* SU, *D* SU, *E* SU, respectively. Each GB at the misorientation angle between *θ* = 50.48° and *θ* = 109.47° results from a combination of *C* SU and *D* SU. Likewise, the misorientation angle between *θ* = 109.47° and *θ* = 141.06° results from a combination of *D* SU and *E* SU. Taking the *θ* = 135.99° boundary as an example, there are two *E* SU and three *D* SU cycles to build the basic GB structure.

**Figure 3:**
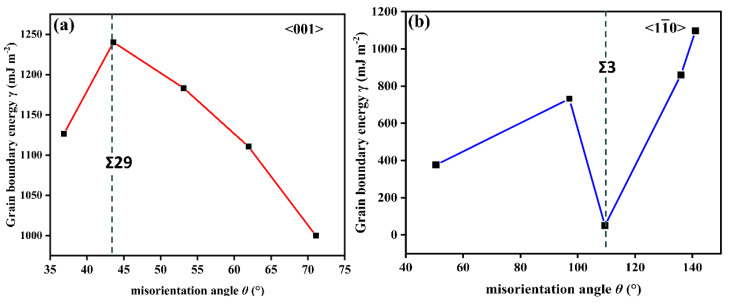
GB energy as a function of misorientation angle for (**a**) 〈001〉 and (**b**) $\langle 1\bar{1}0\rangle$ STGBs. The GB energy for Ni is represented by the black square. The vertical dashed line indicates the GB of interest.

Figure 3 shows the GB energy as a function of misorientation angle for 〈001〉 and $\langle 1\bar{1}0\rangle$ STGBs. Based on previous experiments [46] and simulation studies [47], it has been shown that there is a strong correlation between GB energy and GB structure. In Figure 3b, when the misorientation angle is *θ* = 109.47°, Σ3 ($1\;1\;\bar{1}$) STGB has the lowest GB energy, corresponding to specific structural models. It is clear from Figure 2h that Σ3 ($1\;1\bar{\;1}$) GB has a line defect structure, typically known as a coherent twin boundary. Such a GB formed along the dense plane has a lower energy, which is in accordance with a previous finding by Wolf and Philpot [48].

### 3.2. Hydrogen Segregation at GBs

**Figure 4:**
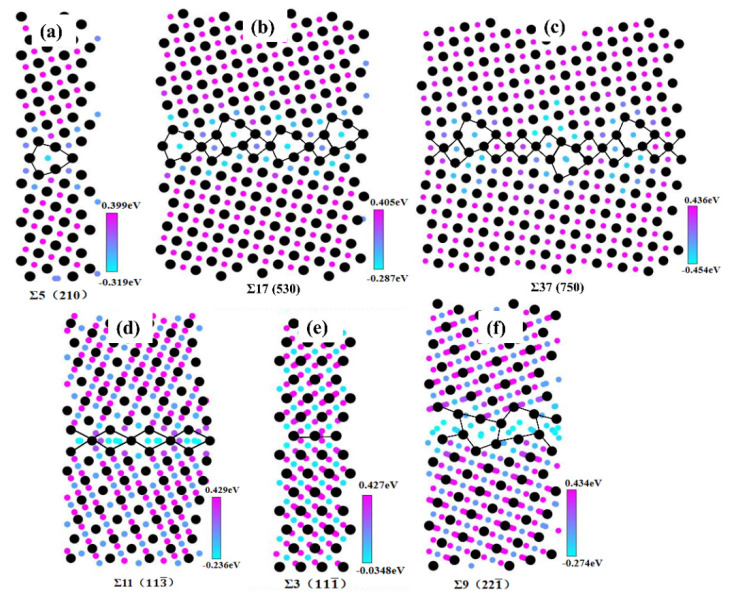
Hydrogen segregation energy diagrams for (**a**–**c**) 〈001〉 and (**d**–**f**) $\langle 1\bar{1}0\rangle$ STGBs.

A controversial area in comprehending HE occurrence is the role of GBs in the diffusion and trapping of H in FCC materials. Based on the GB structures identified in Figure 2, Figure 4 shows the H segregation energy diagrams for six typical GBs; the colored spheres stand for the possible sites where H atoms could be confined, while the black spheres represent Ni atoms. The H segregation energy diagrams at different GBs are variable because the structure of each GB is distinct. When comparing Figure 4d,f, it is clear that the Σ9 ($2\;2\bar{\;1}$) STGB traps more H atoms than the Σ11 ($1\;1\bar{\;3}$) STGB because the E SU has a larger free volume than the C SU. It is worth noting that for the Σ3 ($11\bar{1}$) STGB, essentially no H atoms are segregated at the boundary, and the H segregation energy is negligibly small. In contrast, the other STGBs possess multiple trapping sites and low value of H segregation energy.

**Figure 5:**
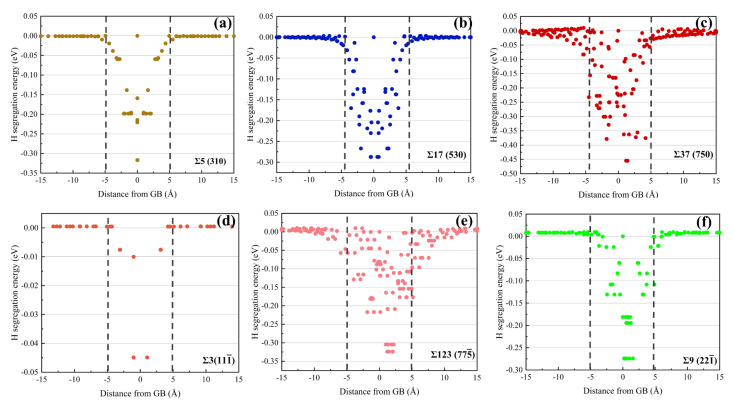
Hydrogen segregation energy distribution as a function of GB distance for (**a**–**c**) 〈001〉 and (**d**–**f**) $\langle 1\bar{1}0\rangle$ STGBs.

To find out the distribution of H segregation to STGBs, Figure 5 manifests that for the majority of GBs, possible H trapping sites with energies less than 0 eV are located within a region ±5 Å from the boundary planes. In the region away from the GBs, the segregation energy approaches 0 eV, being suggestive of a tendency for H diffusion from the bulk into the GBs. This observation agrees well with previous studies [49,50], which reported that some stable FCC structures such as Ni exhibited preferential trapping of H atoms at GBs. Recent experiments using atom probe tomography [25,51] also identified such H retention or trapping at GBs in metals.

### 3.3. Effects of Hydrogen on the Dislocation Nulceaiton and Plastic Deformation of GBs

#### 3.3.1. Dislocation Nucleation Strength Influenced by Hydrogen in GB Model

The mechanical behavior and deformation mechanisms of polycrystalline metals largely depend on the characteristics of their defects or interfaces [52]. In the present study, each of the established bicrystal models was stretched along the Y-axis direction. All curves exhibit a series of stress decreases corresponding to dislocation nucleation. Specifically, the first stress drop is related with the onset of dislocation plasticity; therefore, the highest stress just prior to the first stress drop is referred to as the yield stress.

**Figure 6:**
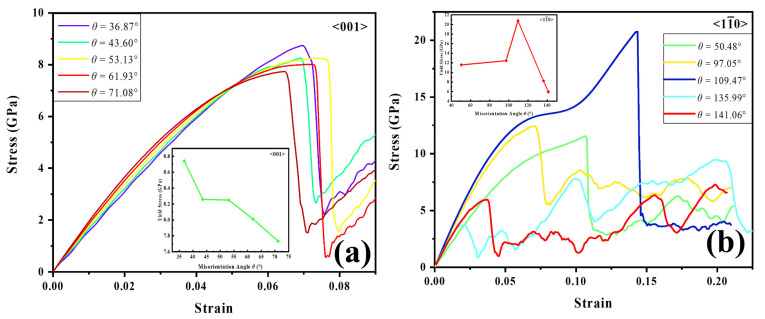
Stress-strain curves for (**a**) 〈001〉 and (**b**) $\langle 1\bar{1}0\rangle$ STGBs. The insets show the dependency of yield stress on misorientation angle.

In order to reveal the connection between the misorientation angle and yield stress, Figure 6 shows the strain-stress curves of selected STGBs and the curves of misorientation angle of GBs with their corresponding yield stress. The yield stress of bicrystal models declines with increasing misorientation angle for 〈001〉 STGBs. On the other hand, for $\langle 1\bar{1}0\rangle$ STGBs, two situations are presented: the yield stress increases with an increasing angle, while the stress in the bicrystal model abruptly reduces when the GB misorientation angle is beyond 109.47°. It can be observed that the yield stress of the Σ123 ($7\;7\;\bar{5}$) and Σ9 ($2\;2\;\bar{1}$) STGBs is lower than other GBs. Such a reduced yield stress can be linked with the E SUs [53].

**Figure 7:**
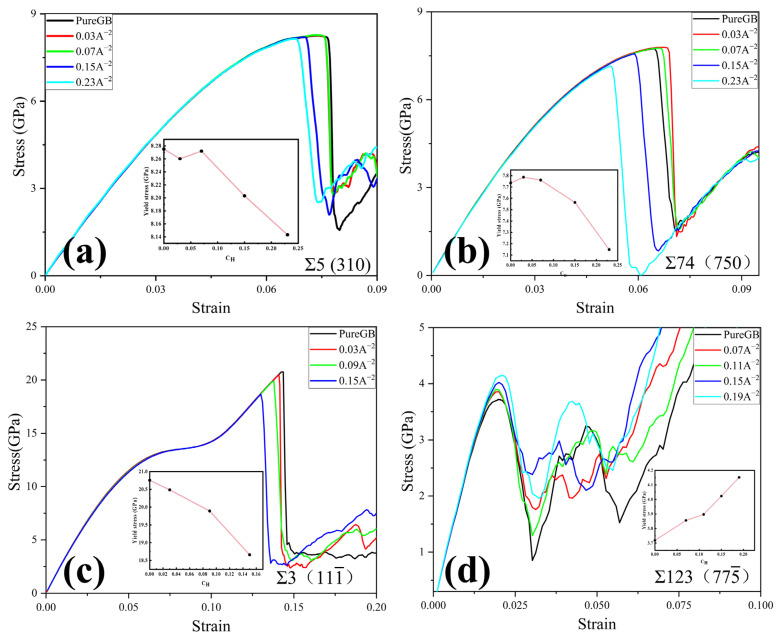
Stress-strain curves for (**a**,**b**) 〈001〉 and (**c**,**d**) $\langle 1\bar{1}0\rangle$ STGBs with various hydrogen concentrations. The insets show the dependency of yield stress on hydrogen concentration.

To further investigate the effect of segregated H on the yield stress of different STGBs, the stress-strain curves of selected STGBs for two tilt axes at different H concentrations are shown in Figure 7. It is clear that, for 〈001〉 STGBs, the yield stress reduces with increasing H concentration. For $\langle 1\bar{1}0\rangle$ STGBs, the stress-strain curves vary in the Σ3 ($1\;1\;\bar{1}$) STGB and Σ123 ($7\;7\;\bar{5}$) STGB. The Σ3 ($1\;1\;\bar{1}$) STGB show a gradual decrease in the yield stress with increasing H concentration; however, Σ123 ($7\;7\;\bar{5}$) STGB manifests an increase in the yield stress. As mentioned above, such a difference in stress variation may be attributable to the aggregation of H atoms within the GB structure and the modification of the GB structure pattern during the stretching process.

#### 3.3.2. The Influence of Hydrogen Segregation on Dislocation Nucleation of 〈001〉 STGB

**Figure 8:**
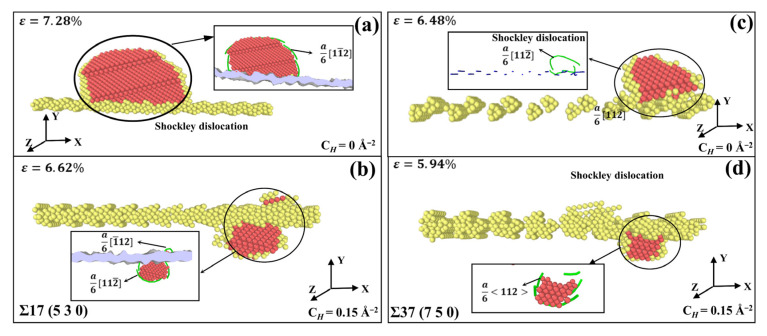
MD snapshots of dislocation nucleation from Ni bicrystal with (**a**,**b**) Σ17 (5 3 0) STGB and (**c**,**d**) Σ37 (7 5 0) STGB without and with H.

In Figure 8, the exact heterogeneous dislocation nucleation (HDN) mechanism can be seen during tensile deformation of the Σ17 (5 3 0) STGB and Σ37 (7 5 0) STGB without and with H, respectively. The HDN mechanism operates via dislocation nucleation from the GBs, and dislocation loops made up of single Shockley dislocations subsequently slide into the grains.

**Figure 9:**
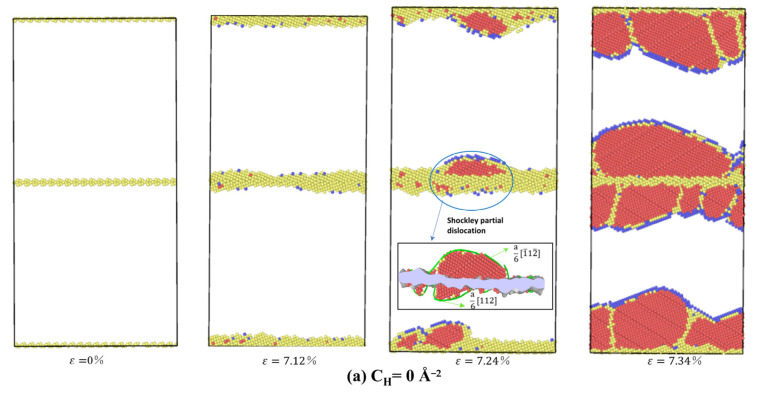

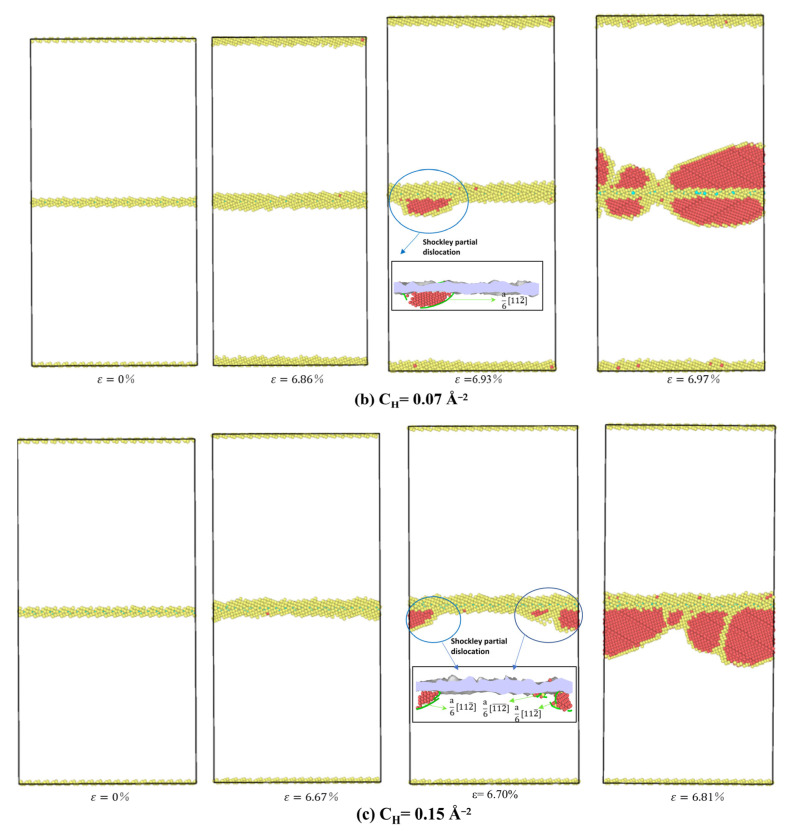
MD snapshots of dislocation nucleation from Ni bicrystal with Σ5 (3 1 0) STGB for various H concentrations. Only the defective arrangement of atoms is shown.

To further investigate the details of dislocation nucleation, the Σ5 (3 1 0) STGB at different deformation stages for various H concentrations is taken as an example. In Figure 9a, when the strain reaches ε= 7.24%, partial dislocation loops with edge and screw features concurrently form at the top and bottom of the GB. In light of DXA analysis, Shockley partial dislocations with the Burge vectors of $\frac{a}{6}\left[112\right]$ and $\frac{a}{6}\left[11\bar{2}\right]$ originate from the GB and propagate along the (111) and (11$\bar{1}$) planes, respectively. With increasing tensile stress, more dislocations gradually and continuously nucleate from the GB plane at a strain of ε = 7.34%.

Figure 9b shows the MD snapshots of Σ5 (3 1 0) STGB at C_H_ = 0.07Å^−2^. In contrast to the H-free case, the boundary structure is significantly disordered, evidenced by a thicker boundary plane. Furthermore, it is clear that the dislocation plasticity occurs earlier; $\frac{a}{6}\left[11\bar{2}\right]$ dislocation nucleates at a strain of ε = 6.93%. This can be attributed to that H addition disorders the boundary structure and increases the strain energy, which promotes dislocation nucleation events [33]. As the tensile strain further increases, a series of dislocations slide continuously into the upper and lower grain regions, corresponding to the plastic deformation process.

In Figure 9c, when the concentration of H is increased to C_H_ = 0.15 Å^−2^, dislocation nucleation becomes earlier, leading to a lower yield strain of ε = 6.70% and a reduced yield stress in Figure 7a. It is worth noting that dislocation events can only be observed in the lower grain, different from the H-free and C_H_ = 0.07Å^−2^ cases. It may be due to that the trapped H leading to an asymmetric GB structure, in which partial dislocations with Burger vectors of $\frac{a}{6}\left[11\bar{2}\right]$, $\frac{a}{6}\left[112\right]$ and $\frac{a}{6}\left[\bar{112}\right]$ are nucleated and emitted into the lower grain.

Overall, the results show that the prominent deformation mechanism of <0 0 1> STGBs is the HDN. H segregation disorders the GB structure and promotes the HDN event within the framework of HELP mechanism. This observation directly explains why the yield stress of <0 0 1> STGBs is reduced with increasing H concentration.

#### 3.3.3. The Influence of Hydrogen Segregation on Dislocation Nucleation of $\langle 1\bar{1}0\rangle$ STGB

**Figure 10:**
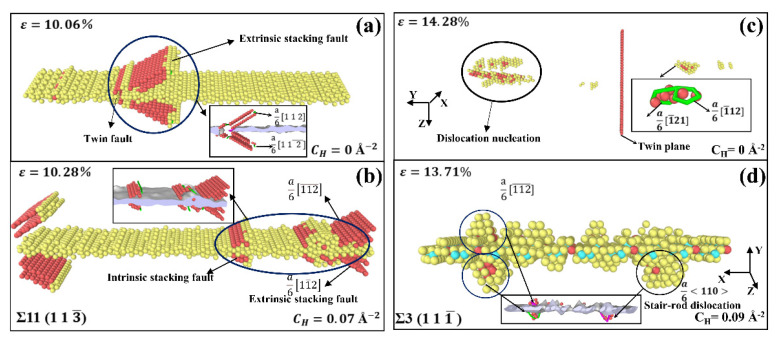
MD snapshots of dislocation nucleation from Ni bicrystal with (**a**,**b**) Σ11 ($1\;1\;\bar{3}$) STGB and (**c**,**d**) Σ3 ($1\;1\;\bar{1}$ ) STGB without and with H.

**Figure 11:**
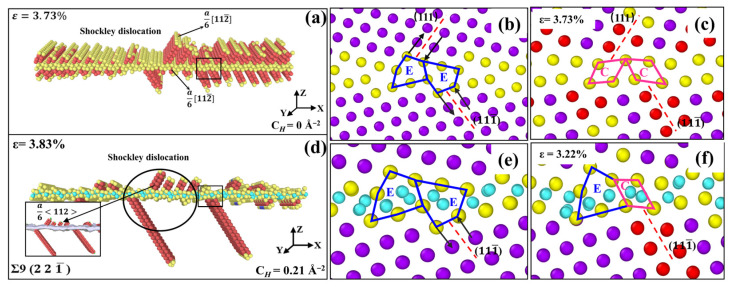
MD snapshots of dislocation nucleation from Ni bicrystal with Σ9 ($2\;2\;\bar{1}$) STGB without and with H. (**b**,**c**,**e**,**f**) Enlarged images for nucleation region of the boundary marked in (**a**,**d**), respectively.

The results of MD simulations for several $\langle 1\bar{1}0\rangle$ STGBs are shown in Figure 10 and Figure 11. Figure 10a,b depict the dislocation nucleation process of Σ11 ($1\;1\;\bar{3}$) STGB at 10K. The structure of Σ11(1 1$\;\bar{3}$) STGB comprises a straightforward arrangement of C SUs with a relatively low GB energy value, making its nucleation mechanism quite special. The dislocations begin to form at the GB and slide along (111) and (11$\bar{1}$) planes into the upper and lower grains when the yield point is reached at ε = 10.06%, leaving behind a series of extrinsic stacking fault. In the presence of H, as depicted in Figure 10b, some dislocations with Burgers vectors of $\frac{a}{6}\left[\bar{112}\right]$ and $\frac{a}{6}\left[1\bar{1}2\right]$ tend to appear, and both intrinsic and extrinsic stacking fault begin to form at the GB. The occurrence of intrinsic stacking fault can be associated with the fact that H atoms affect the structural alteration of the GB, which obstructs the conversion of intrinsic stacking fault to external stacking fault.

For the Σ3 ($1\;1\;\bar{1}$) STGB, the GB energy is the lowest of all $\langle 1\bar{1}0\rangle$ STGBs. As it has a simple GB structure, there is no extra free volume that can accommodate dislocations. As seen in Figure 10c, dislocations are nucleated from the grains in the form of dislocation loops, and there are no dislocations nucleating from the GB plane. Conversely, in the presence of H, as shown in Figure 10d, the local GB atoms change, and the arrangement of GB atom becomes increasingly disordered. The change in GB structure caused by the presence of H triggers dislocation nucleation from the boundary plane, with a series of Shockley dislocations and stair-rod dislocations nucleating at the GB when the yield stress is reached.

As mentioned previously, the Σ9 ($2\;2\;\bar{1}$) STGB is comprised of E SUs. Because of the large free volume in E SUs, there is a specific structural deformation during dislocation nucleation. As shown in Figure 11a, the dislocations are nucleated from the GB, and a series of $\frac{a}{6}\langle 112\rangle$ Shockley partial dislocations propagate on the (111) and (11$\bar{1}$) planes. The nucleation event is accompanied by a transformation of E SUs into C SUs, as shown in Figure 11b,c. When considering the presence of H, Figure 11d shows that dislocation nucleation is retarded, and only several dislocations propagate from the GB. Atomic configurations reveal that H atoms segregated at the GB suppress the collapse of E SUs, thus hindering the nucleation event.

## 4. Summary

In the present study, H segregation and trapping at various 〈001〉 and $\langle 1\bar{1}0\rangle$ bicrystal models with STGBs were identified using MD simulations. The effect of H on dislocation nucleation and plastic deformation of STGBs was subsequently investigated. Several conclusions are drawn as follows.

(1) The ability of the GB to trap H atoms is very sensitive to the GB structure and GB energy. The GBs with higher GB energy own a larger free volume, thus trapping more H atoms. There are essentially no H atoms segregating at the Σ3 ($11\bar{1}$) STGB, while for other STGBs, H atoms are distributed within a region ±5 Å from the boundary planes.

(2) For 〈001〉 STGB, the plastic deformation mechanism is dominated by HDN. H segregation disorders the GB structure and promotes the HDN event, leading to a reduced yield stress with increasing H concentration.

(3) For $\langle 1\bar{1}0\rangle$ STGB, the yield stress is increased or decreased as the H concentration increases, depending on the GB structure. The reduced stress is associated with the H atoms disordering the boundary structure, while the increased stress is attributed to the fact that the presence of H inhibits the structural transformation and dislocation nucleation.

There are strong links between our simulation results and experimental observations. Analysis of stress–strain curves generated from uniaxial tension of 〈001〉 STGBs indicates that the introduction of H causes a reduction in the yield stress required for dislocation nucleation from the boundary. This is in accordance with experimental observations of H-enhanced dislocation generation [54,55] as well as slip transfer across GBs [2,56,57]. In addition, it is found that H-segregated GBs tend to hold in their initial configurations and suppress structural evolution or predissociation, as shown in Figure 11. This observation was revealed by Ferreira et al. [58], who demonstrated that solute H could stabilize the dislocations and stop the evolution of structural configurations. These findings can provide a new perspective towards understanding the experimentally-observed HE in metals.

## Figures and Tables

**Table 1:** Characterization of all considered GBs, including the model size, misorientation angle (*θ)*, GB structure unit, and GB energy γ ($\mathrm{mJ}\cdot m^{-2}$).

GB Type	Angle	Model Size	GB Structure	GB Energy
	*θ* (°)	X × Y × Z (nm)		$\gamma \;(mJ\cdot m^{-2})$
Σ5 (3 1 0)〈001〉	36.87°	11.13 × 22.26 × 3.52	*|DBDB|*	1126.25
Σ29 (5 2 0)〈001〉	43.60°	15.16 × 22.75 × 3.52	*|BBD|*	1240.23
Σ5 (2 1 0)〈001〉	53.13°	15.74 × 22.04 × 3.52	*|BBB|*	1183.13
Σ17 (5 3 0)〈001〉	61.93°	16.42 × 24.63 × 3.52	*|BABA|*	1110.48
Σ37 (7 5 0)〈001〉	71.08°	15.14 × 24.24 × 3.52	*|BAAABAA|*	999.80
Σ11 ($1\;1\;\bar{3}$)$\langle 1\bar{1}0\rangle$	50.48°	13.21 × 23.35 × 4.98	*|CC|*	375.83
Σ57 (4 4$\;\bar{5}$)$\langle 1\bar{1}0\rangle$	97.05°	18.79 × 26.58 × 4.98	*|CD|*	731.83
Σ3 ($1\;1\;\bar{1}$)$\langle 1\bar{1}0\rangle$	109.47°	16.38 × 23.17 × 4.98	*|DDDD|*	50.38
Σ123 (7$\;7\;\bar{5}$)$\langle 1\bar{1}0\rangle$	135.99°	11.04 × 23.42 × 4.98	*|EDDED|*	860.61
Σ9 (2 2$\;\bar{1}$)$\langle 1\bar{1}0\rangle$	141.06°	11.95 × 23.23 × 4.98	*|EE|*	1097.45

## Data Availability

The data presented in this study are available on request from the corresponding author.

## Nomenclature

GB	grain boundary
STGBs	symmetrical tilt grain boundaries
HDN	heterogeneous dislocation nucleation
HE	hydrogen embrittlement
IHE	internal hydrogen embrittlement
EHE	environmental hydrogen embrittlement
HEDE	hydrogen-enhanced decohesion
HELP	hydrogen enhanced localized plasticity
MD	molecular dynamics
EAM	embedded-atom method
LAMMPS	large-scale atomic/molecular massively parallel simulator
NPT	isothermal–isobaric ensemble
CNA	common neighbor analysis
CSP	centro-symmetry parameter
DXA	dislocation extraction algorithm
OVITO	open visualization tool
